# The impact of childhood acute rotavirus gastroenteritis on the parents’ quality of life: prospective observational study in European primary care medical practices

**DOI:** 10.1186/1471-2431-12-58

**Published:** 2012-05-31

**Authors:** Javier Diez Domingo, Marian Patrzalek, Luigi Cantarutti, Benoit Arnould, Juliette Meunier, Montse Soriano-Gabarro, Nadia Meyer, Jean-Yves Pirçon, Katsiaryna Holl

**Affiliations:** 1 Centro Superior de Investigacion en Salud Publica (CSISP), Area de Investigación en Vacunas, Avda Catalunya 21, Valencia, 46020, Spain; 2 Nzoz Promed, Pediatric Clinic, Kielce, Poland; 3 Pedianet network, Padova, Italy; 4 MAPI Consultancy, Lyon, France; 5 Bayer Schering Pharma AG, Berlin, Germany; 6 GlaxoSmithKline Biologicals, Wavre, Belgium

**Keywords:** Rotavirus, Gastroenteritis, Paediatric, Quality of life

## Abstract

**Background:**

Rotavirus (RV) is the commonest cause of acute gastroenteritis in infants and young children worldwide. A Quality of Life study was conducted in primary care in three European countries as part of a larger epidemiological study (SPRIK) to investigate the impact of paediatric rotavirus gastroenteritis (RVGE) on affected children and their parents.

**Methods:**

A self-administered questionnaire was linguistically validated in Spanish, Italian and Polish. The questionnaire was included in an observational multicentre prospective study of 302 children aged <5 years presenting to a general practitioner or paediatrician for RVGE at centres in Spain, Italy or Poland. RV infection was confirmed by polymerase chain reaction (PCR) testing (n = 264). The questionnaire was validated and used to assess the emotional impact of paediatric RVGE on the parents.

**Results:**

Questionnaire responses showed that acute RVGE in a child adversely affects the parents’ daily life as well as the child. Parents of children with RVGE experience worry, distress and impact on their daily activities. RVGE of greater clinical severity (assessed by the Vesikari scale) was associated with higher parental worries due to symptoms and greater changes in the child’s behaviour, and a trend to higher impact on parents’ daily activities and higher parental distress, together with a higher score on the symptom severity scale of the questionnaire.

**Conclusions:**

Parents of a child with acute RVGE presenting to primary care experience worry, distress and disruptions to daily life as a result of the child’s illness. Prevention of this disease through prophylactic vaccination will improve the daily lives of parents and children.

## Background

Rotavirus infection is the leading cause of acute gastroenteritis in infants and young children worldwide [1], and is estimated to cause over 146,000 hospital admissions per year in children aged <5 years in the World Health Organization (WHO) European region [2]. In the 23.6 million children aged <5 years in the European Union (EU) countries, 3.6 million episodes of rotavirus illness are estimated to occur annually, resulting in almost 700,000 outpatient visits, over 87,000 hospitalisations and 231 deaths per year [3].

Vaccination against rotavirus is recommended in guidelines issued by the European Society for Paediatric Infectious Diseases and European Society for Paediatric Gastroenterology, Hepatology, and Nutrition [4,5], and by the WHO Strategic Advisory Group of Experts [6]. Two rotavirus vaccines have demonstrated good clinical efficacy and safety in large clinical trials and are currently available in Europe, a two-dose live attenuated monovalent human vaccine (GSK Biologicals, Rixensart, Belgium) [7,8] and a three-dose live human-bovine reassortant vaccine (Sanofi Pasteur MSD) [9].

Rotavirus gastroenteritis is associated more frequently with severe symptoms and increased hospital admissions, compared with acute gastroenteritis due to other infectious causes [10]. The severity of symptoms may cause worry, anxiety and distress in the affected child’s parents, as well as distress to the child. Family life may also be disrupted, for example if parents have to take time away from work to look after a child with rotavirus gastroenteritis or to travel to healthcare facilities, or if parents’ other daily activities are curtailed. Information about the emotional impact of rotavirus gastroenteritis on the family is important for a fuller understanding of the burden of rotavirus disease. We conducted a quality of life study as part of the **S**urveillance for **P**ractitioner/Paediatrician for **R**otavirus **I**nfections in **K**ids (**SPRIK**) epidemiological study assessing the disease burden of rotavirus among children seen by primary care physicians, to investigate the impact of a child’s rotavirus gastroenteritis on the quality of life of parents and children in three European countries, namely Spain, Italy and Poland. To our knowledge, this is the first multicentre study to investigate this issue using the same validated questionnaire in multiple countries.

## Methods

### Study design

This analysis was conducted as part of the **S**urveillance for **P**ractitioner/Paediatrician for **R**otavirus **I**nfections in **K**ids (**SPRIK**) study. The main SPRIK study was an observational prospective multicentre study assessing the burden of rotavirus infections in children presenting to general practitioners or paediatricians in six European countries, and has been fully published elsewhere [11]. Children aged less than 5 years were eligible for the study if they presented to participating general practitioners or paediatricians with acute rotavirus gastroenteritis (rotavirus-positive stool sample) and their parent/guardian gave written informed consent. Acute rotavirus gastroenteritis was defined as diarrhoea for less than 14 days, with or without vomiting. Diarrhoea was defined as three or more looser than normal stools within a 24-hour period. Stool samples were initially tested for rotavirus by the general practitioner or paediatrician using a rapid immunochromatographic test kit (RotaStrip®, Coris BioConcept, Gembloux, Belgium) at the initial visit or within 4 days. Rotavirus-positive samples were confirmed and characterised by polymerase chain reaction (PCR) testing at a central reference laboratory (Health Protection Agency laboratory, London, UK) using published oligonucleotide primers and methods [12,13]. As all cases enrolled in the study were RotaStrip®-positive, the study did not have a rotavirus-negative control group, although PCR-negative cases were analysed as a comparison with the PCR-positive cases. The clinical severity of rotavirus gastroenteritis was categorised according to the Vesikari score [14]. As testing for rotavirus infection was not part of routine clinical practice, it was not possible to determine whether patients enrolled in the study had a previous history of rotavirus gastroenteritis. The study was carried out before widespread introduction of rotavirus vaccines, and therefore few if any children in the study population had received rotavirus vaccination.

The study was conducted in accordance with the Declaration of Helsinki and Good Clinical Practice guidelines. The protocol was reviewed and approved by the local independent ethics committee or institutional review board for each participating general practitioner/paediatrician network (*Italy*: Independent Ethics Committee for the Assessment of Clinical trials on Drugs of Local Healthcare Authority No. 8, Asolo; *Spain*: Primary Health-Care Clinical Review Board for Valencia and Castellón, Valencia; *Poland*: Bioethics Committee, Regional Medical Chamber, Kielce).

The present study on quality of life was conducted among SPRIK participants enrolled at centres in Spain, Poland and Italy. Parents or caregivers of the enrolled children were interviewed about their child’s symptoms, and at the end of the study visit a copy of a self-administered questionnaire was given to the parent of each enrolled patient. Parents were asked to complete the questionnaire at home within seven days of the clinic visit and to return the completed questionnaire using a prepaid envelope or by handing it to their paediatrician. During a follow-up phone call or visit, a study nurse checked whether the questionnaire had been completed and returned, and reminded the parents to return it if they had not already done so.

The questionnaire was developed from an initial 54-item pilot questionnaire, linguistically validated in Spanish, Polish and Italian. After multitrait analysis and item reduction, the final questionnaire consisted of 44 items in five scales, Symptom Severity (13 items); Child’s Behaviour (6 items); Parents’ Worries due to Symptoms (8 items); Parents’ Distress (7 items); and Impact on Parents’ Daily Activities (10 items). The questionnaire was validated by assessing internal consistency reliability, floor and ceiling effects and scale-scale correlations, as described in Additional file 1 and presented at the International Society for Pharmacoeconomics and Outcomes Research (ISPOR) congress in 2008 [15].

### Statistical analysis

Analyses of the scores were carried out on data from the cohort of patients with a rotavirus-positive PCR test. Descriptive statistics including frequency, mean, and standard error of the mean were recorded for the quantitative variables. For qualitative variables, number and percentage were recorded, with missing data included in the calculation of percentages. Non-parametric or ordinal variables were compared using the Mann–Whitney-Wilcoxon test when comparing two groups, or the Kruskal-Wallis test when comparing three or more groups. All data processing was performed using SAS for Windows (Statistical Analysis System, Version 9).

## Results

### Demographic and clinical characteristics of the study population

Questionnaires were issued to the parents of all RotaStrip®-positive subjects, 426 in total, of whom 213 were in Spain, 126 in Italy and 87 in Poland. Subjects were enrolled at 15 participating centres in Spain, 12 in Italy and 15 in Poland. A total of 302 respondents returned the questionnaire with at least one item completed, 190 in Spain, 37 in Italy and 75 in Poland. The response rate was 302/426 (71%) overall, 190/213 (89%) in Spain, 75/87 (86%) in Poland, and 37/126 (29%) in Italy. Of the 302 questionnaires returned, the majority of cases (87%, n = 264) were rotavirus-positive on the PCR test, and 11% (n = 34) were rotavirus-negative on PCR. PCR results were missing in 4 cases.

Questionnaires from parents of children with PCR-confirmed rotavirus gastroenteritis were included in the main analysis (n = 264). Demographic and clinical characteristics for this population are presented in Table 1. The sex ratio was balanced between male and female subjects (n = 135 and n = 129, respectively). One hundred and seventy-one parents (65%) were Spanish, 67 (25%) were Polish and 26 (10%) were Italian. The majority of the patients (n = 199, 75%) had no underlying major medical history. A total of 84 patients (32%) had attended a day-care centre and 9 patients (3%) had been admitted to a hospital in the 14 days prior to the visit. Clinical symptoms were categorised as mild on the Vesikari scale in 70 patients (27%), moderate in 137 (52%) and severe in 57 (22%) (Table 1).

**Table 1:** Demographic and clinical characteristics of study subjects with PCR-confirmed rotavirus gastroenteritis (n = 264)

**Parameter**	**Number**	**%**	
Age, months:			
0–5	23	9	
6–11	73	28	
12–23	99	37	
24–35	33	12	
36–59	36	14	
Male	135	51	
Female	129	49	
Country:			
Spain	171	65	
Poland	67	25	
Italy	26	10	
**Medical history of the patient**	**N, answering yes**	**%, answering yes**	
Prematurity	12	5%	
Cardiac disease	6	2%	
Pulmonary disease	31	12%	
Immunodeficiency	1	<1%	
Other disease	29	11%	
No disease	199	75%	
**Risk factors**	**N, answering yes**	**%, answering yes**	
Hospital admission within 14 days prior to enrolment	9	3%	
Attending day-care	84	32%	
**Severity as assessed by Vesikari score, by country**			
	**Mild, n (%)**	**Moderate, n (%)**	**Severe, n (%)**
Spain	47 (27%)	78 (46%)	46 (27%)
Poland	17 (25%)	44 (66%)	6 (9%)
Italy	6 (23%)	15 (58%)	5 (19%)
Total	70 (27%)	137 (52%)	57 (22%)

A further 34 questionnaires were received from parents of children who were rotavirus-negative on PCR testing. PCR results were missing in 4 cases.

### Questionnaire validation

Of the 302 questionnaires returned (n = 264 PCR-positive, n = 34 PCR-negative, n = 4 PCR results missing), 291 questionnaires provided sufficient data for inclusion in the analysis of psychometric properties. Item convergent and discriminant validity was good, no strong floor or ceiling effects were observed, internal consistency reliability was high, and scale–scale correlations indicated a strong relationship between all the scales showing consistency but no redundancy. The validation results are presented in more detail in Additional file 1.

### Impact of rotavirus gastroenteritis on children and their parents

**Figure 1:**
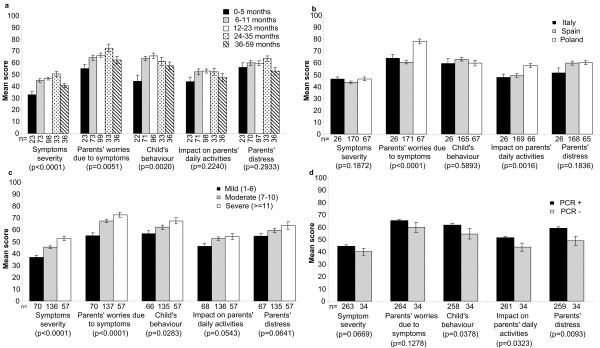
** Questionnaire scores in study subjects.** Questionnaire scores in study subjects with PCR-confirmed rotavirus gastroenteritis (n = 264) by (**a**) age group, (**b**) country and (**c**) severity of symptoms as assessed by Vesikari score. Questionnaire scores by rotavirus status as assessed by polymerase chain reaction (PCR) in study subjects with PCR data (n = 298) (**d**). Mean score with standard error of the mean plotted as error bar.

The Symptom Severity, Parents’ Worries due to Symptoms and Child’s Behaviour scales all showed statistically significant differences across age groups, although the pattern varied by scale (Figure 1a). The Symptom Severity (p < 0.001) and Parents’ Worries due to Symptoms (p < 0.01) scores both increased with the child’s age up to 35 months and then declined (Figure 1a). The Child’s Behaviour score (p < 0.01) was lowest for children aged up to 6 months and highest in children aged 12–23 months, then progressively declined with age. Parents of children aged up to 6 months reported less impact on their daily activities than parents of children in the other age groups, though the difference did not reach statistical significance (p > 0.1). There were no statistically significant differences in the scores between boys and girls on any of the five scales (p > 0.1 for Symptom Severity, Parents’ Worries due to Symptoms, Child’s Behaviour, and Impact on Parents’ Daily Activities), although the Parents’ Distress score tended to be higher for boys than for girls (p = 0.0637).

Figure 1b shows the scores on each scale analysed by country. Polish parents were more worried by the symptoms than parents from Spain and Italy (p < 0.001). Parents from Poland also reported a more severe impact of their child’s rotavirus gastroenteritis on their daily activities, compared with Spanish and Italian parents (p < 0.01). There were no statistically significant differences between countries in the Symptom Severity, Child’s Behaviour or Parents’ Distress scores. These findings suggest that symptoms and behaviour as perceived by parents were the same for all children, regardless of country, but that the impact on the parents in terms of worry or disruption to daily activities varied by country.

Increasing severity of rotavirus gastroenteritis according to the Vesikari score (Figure 1c) was associated with higher parental worry due to symptoms (p < 0.001), greater changes in the child’s behaviour (p < 0.05) and a trend towards greater impact on the parents’ daily activities (p < 0.1) and higher parental distress (p < 0.1), as well as a higher score on the symptom severity scale of the questionnaire (p < 0.001).

Parents who had prior contact with a physician or a medical facility due to the same rotavirus gastroenteritis episode in their child had a higher Parents’ Distress score than parents who had not (p < 0.05), although no statistically significant difference was observed in the other scales of the questionnaire. No statistically significant differences in the scores were observed between children with or without a medical history, or between children who were admitted to a hospital in the previous 14 days (n = 9) and those who were not (data not shown).

Figure 1d shows the scores on each scale for cases of rotavirus gastroenteritis confirmed by PCR (n = 264), compared with cases that tested negative for rotavirus by PCR (n = 34). Rotavirus confirmed by PCR was associated with significantly greater change in the child’s behaviour (p < 0.05), parental distress (p < 0.01) and impact on the parents’ daily activities (p < 0.05), compared with cases with a negative PCR test (Figure 1d). There were no statistically significant differences in the Symptom Severity or Parents’ Worries due to Symptoms scores (Figure 1d).

## Discussion

In this paper we present results from a questionnaire designed to be completed by parents to assess the impact of childhood rotavirus gastroenteritis on affected children and their parents as a quality of life measurement. The questionnaire was validated and used as part of a multinational observational prospective study of children aged <5 years presenting to a general practitioner or paediatrician for acute rotavirus gastroenteritis. To our knowledge, this is the first multicentre multinational study to investigate this issue using the same validated questionnaire in a range of different countries.

Two hundred and sixty-four children with PCR-confirmed rotavirus infection were included in the main analysis. The responses to the self-administered questionnaire showed that PCR-confirmed rotavirus gastroenteritis was associated with changes in the child’s behaviour, parental distress, parental worry and impact on parents’ daily activities. This is consistent with results from previous studies that have found rotavirus gastroenteritis to be associated with particularly severe symptoms [10][16]. The validity of the questionnaire was supported by parents reporting a greater change in their child’s behaviour and more worry due to symptoms when the child had clinically severe rotavirus gastroenteritis than when the child had clinically mild rotavirus gastroenteritis (as assessed by the Vesikari scale).

The five scales showed subtly different patterns of variation in relation to demographic parameters. Parents’ perceptions of symptom severity and their worries due to the symptoms unexpectedly increased with the child’s age up to 35 months and then declined. Conversely, the Child’s Behaviour score was lower for children aged under 6 months than for the older age groups. This may reflect the generally lower level of activity in children aged under 6 months, which could make it more difficult to identify a change in behaviour compared with older children. The impact on parents’ daily activities showed a trend to lower scores in children aged under 6 months compared with the older age groups, possibly because parents of children aged under 6 months are likely to be on parental leave, or possibly because a child aged under 6 months requires a great deal of parental attention regardless of health status, and therefore the change in response to illness may be correspondingly less than in older age groups. Another possibility is that parents of children aged under 6 months may be more likely to visit hospitals or emergency rooms if the child has acute severe rotavirus gastroenteritis symptoms, rather than presenting to general practice. As our study was conducted in a general practice setting, it may thus have tended to include less severe cases in very young children, which in turn may have influenced parents’ assessments of the child’s behaviour and impact on the parents’ daily activities.

Rotavirus gastroenteritis confirmed by PCR testing was associated with significantly greater change in the child’s behaviour (p < 0.05), parental distress (p < 0.01) and impact on the parents’ daily activities (p < 0.05), compared with cases that initially tested positive on the rapid test but were negative on PCR. The rapid test is known to have a lower sensitivity than PCR testing (sensitivity around 87%, specificity around 80% [GlaxoSmithKline Biologicals, unpublished data]). The relatively low specificity would be expected to produce some false-positive results (around 20%), and thus the RotaStrip® rapid test was used as a screen with positive results confirmed using PCR as a reference assay. Most of the cases reported in this analysis (n = 264) were positive on both PCR and the rapid test, and 34 cases that were initially positive on the rapid test were negative on PCR (PCR data were missing in 4 cases). The study was conducted before widespread introduction of rotavirus vaccination, and therefore represents the situation in an unvaccinated population.

Parents in all three countries in the study perceived similar changes in their child’s behaviour and similar symptom severity, but there appeared to be subtle country variations in the parents’ perceptions of worry, distress or disruption to daily activities. Polish parents reported greater levels of worry due to their child’s symptoms and a more severe impact on their daily activities compared with Spanish or Italian parents, and there was a trend to greater parents’ distress in Poland and Spain than in Italy. This did not appear to be explained by differences in clinical severity between countries, as Poland did not have a higher percentage of severe cases than Spain or Italy (Table 1). These differences may indicate cultural or social differences, which could be explored in further research.

These variations in the results between the different scales indicate that the five scales provide complementary information. Thus, we elected not to calculate a global score, as the five individual scores provide a more complete picture of the impact of rotavirus gastroenteritis than a global score could achieve.

Among the study’s strengths are its laboratory confirmation of rotavirus infection using PCR, its assessment of case severity using an objective medical evaluation (the Vesikari score), and its use of a questionnaire validated by recognised methods (as described in Additional file 1). Another key strength of the study is its inclusion of participants from three different countries, applying the same study protocol and design. Participants in all three countries were assessed using the same psychometrically validated questionnaire containing the same items, thereby allowing exploration of similarities and differences in the impact of rotavirus across the different countries and providing a broad perspective on the burden of rotavirus gastroenteritis in Europe.

One limitation of our study is that it was not designed or powered to compare scores according to demographic or risk factors. Thus, although the results indicate that the questionnaire is able to discriminate between different groups of patients, which is encouraging for the future use of the questionnaire in further research, the differences reported here should be interpreted with caution. Another limitation is that the number of respondents in Italy was small. The lower response rate in Italy compared with the other two countries may be due to differences in follow-up, as not all the participating centres in Italy followed up parents who had not returned the questionnaire. It was not possible to determine whether patients enrolled in the study had a previous history of rotavirus gastroenteritis, because testing for rotavirus infection was not part of routine clinical practice when the study was conducted, so we could not assess whether the quality of life impact varied between first and subsequent episodes.

Another limitation of our study is that it did not include a control group of rotavirus-negative patients, as all cases enrolled in the study tested positive for rotavirus on the rapid test. The PCR-negative cases in the study were analysed as a comparison group. However, the comparison should be interpreted with caution, as the PCR-negative cases were few (n = 34). A full comparison of the impact of rotavirus gastroenteritis with that of non-rotavirus gastroenteritis would require a different study design with a rotavirus-negative control group. Our results suggest that the questionnaire described here would be a suitable instrument for such a study.

Our results are broadly consistent with those of other published studies that have investigated the impact of rotavirus gastroenteritis on families. In Canada, a prospective study in children aged under 3 years presenting in outpatient settings with rotavirus gastroenteritis (the MIRAGE study) found that over half (53.8%) of parents of children with rotavirus gastroenteritis had to take time off work as a result of their child’s illness [17]. A sub-analysis of the same study used the EuroQol 5D (EQ-5D) to assess the parents’ quality of life, and reported adverse effects of the child’s rotavirus gastroenteritis on the parents’ EQ-5D scores on the dimensions of anxiety/depression, pain/discomfort and usual activities [18]. These studies are consistent with our findings of adverse effects of childhood rotavirus gastroenteritis on parental worry, distress and impact on daily activities. The EQ-5D scale is widely used [19] and can be converted into utility values using a country-specific algorithm [20], but it is a generic quality of life instrument rather than a disease-specific measure. A smaller study of children aged 2–36 months presenting with rotavirus gastroenteritis at outpatient clinics or emergency rooms in North Carolina, USA, used qualitative interviews with 17 parents to explore the emotional impact of the child’s illness on the family [21]. The parents were frightened and worried by the severity of the symptoms, and reported that the child’s illness caused loss of sleep, missed work and difficulty in completing normal household tasks [21]. Although this study did not attempt to quantify these adverse emotional and practical effects using a scoring system, the issues reported are consistent with those addressed by our questionnaire.

In Europe, a study in 25 hospitals and five primary care centres in Spain found that rotavirus-positive gastroenteritis in children aged under 2 years was associated with more severe parental worry and more disruption to household tasks than rotavirus-negative gastroenteritis [22]. The multi-country REVEAL study conducted in Belgium, France, Germany, Italy, Spain, Sweden and the UK in 2004–2005 found high levels of self-reported parental stress among parents of children with rotavirus gastroenteritis aged under 5 years, with mean stress levels of 4.5 to 8.99 on a visual analogue scale from 1 (no stress) to 10 (extremely stressed) [23]. A large survey conducted in 69 paediatric practices in Germany in 2005–2006 used a questionnaire developed for the study (the Parental Appraisal of the Morbidity of Diarrhea in Infants and toddlers [PAMODI]) to assess clinical and behavioural symptoms, treatment and parental feelings in 2023 parents of children aged under 2 years presenting with diarrhoea [24]. Parents’ feelings were characterised by sympathy and anxiety for the child, followed by feelings of helplessness, stress and exhaustion. Over half (53.6%)of parents of children with severe gastroenteritis reported restrictions on their normal daily activities [24]. The validity of the PAMODI questionnaire was supported by the finding that clinically more severe disease (measured by a modified Vesikari score) was associated with a higher perceived disease burden, but the psychometric properties of the PAMODI questionnaire were not formally investigated. Our study builds on the earlier results by using a fully validated questionnaire specifically designed for use with parents of children experiencing rotavirus gastroenteritis.

## Conclusions

The present study demonstrates that childhood rotavirus gastroenteritis presenting to primary care imposes an important emotional and practical burden on the parents of the affected child, causing worry, distress and disruption to normal daily activities. This adverse impact on parents’ quality of life contributes to the overall disease burden of rotavirus gastroenteritis. Prevention of this disease through prophylactic vaccination will improve the daily lives not just of the children, but of their families.

## Abbreviations

EQ-5D: EuroQol 5D; EU: European Union; ISPOR: International Society for Pharmacoeconomics and Outcomes Research; PAMODI: Parental Appraisal of the Morbidity of Diarrhea in Infants and toddlers; PCR: Polymerase chain reaction; RV: Rotavirus; RVGE: Rotavirus gastroenteritis; SPRIK: **S**urveillance for **P**ractitioner/Paediatrician for **R**otavirus **I**nfections in **K**ids; WHO: World Health Organization.

## Competing interests

Dr J Diez Domingo is a member of advisory boards and speaker bureaus for GlaxoSmithKline, SPMSD and Pfizer for which he receives payment. He is also a principal investigator in clinical trials for GlaxoSmithKline and SPMSD.

Dr L Cantarutti does not have any conflicts of interest to declare.

Dr M Patrzalek received payment for services on lecture bureaus for GlaxoSmithKline, Polpharma, and Pfizer.

Dr Benoit Arnould and Juliette Meunier work for MAPI Values who receive grants and payment from GlaxoSmithKline Group of Companies to conduct statistical analyses and research.

Nadia Meyer, Jean-Yves Pircon and Katsiaryna Holl are employed by the GlaxoSmithKline Group of Companies. Montse Soriano Gabarro was an employee of GlaxoSmithKline Group of Companies at commencement of this study.

This study, including preparation of the manuscript, was funded by GlaxoSmithKline Biologicals, Rixensart, Belgium. The study sponsor was involved in the design and conduct of the trial, and in data collection and analysis. All authors had full access to the clinical trial report and reviewed all drafts of the manuscript. The corresponding author had final responsibility for the decision to submit for publication.

## Authors’ contributions

JDD, LC, MP, MSG, NM and JYP, took part in the design of the study and collection of the data. JYP, JM and BA performed the data analyses. All authors reviewed and commented on the draft manuscript, and all authors read and approved the final manuscript.

## Pre-publication history

The pre-publication history for this paper can be accessed here:

http://www.biomedcentral.com/1471-2431/12/58/prepub

## Supplementary Material

Additional file 1 **Questionnaire validation.** Details of the validation of the questionnaire in DOC format. Click here for file

## References

[B1] Parashar UD Bresee JS Gentsch JR Glass RI Rotavirus Emerg Infect Dis 1998 4 561 570 10.3201/eid0404.980406 9866732 PMC2640254

[B2] Williams CJ Lobanov A Pebody RG Estimated mortality and hospital admission due to rotavirus infection in the WHO European region Epidemiol Infect 2009 137 607 616 10.1017/S0950268808001714 19134232

[B3] Soriano-Gabarro M Mrukowicz J Vesikari T Verstraeten T Burden of rotavirus disease in European Union countries Pediatr Infect Dis J 2006 25 S7 S11 10.1097/01.inf.0000197622.98559.01 16397431

[B4] Vesikari T Van Damme P Giaquinto C Gray J Mrukowicz J Dagan R Guarino A Szajewska H Usonis V European Society for Paediatric Infectious Diseases/European Society for Paediatric Gastroenterology, Hepatology, and Nutrition evidence-based recommendations for rotavirus vaccination in Europe: executive summary J Pediatr Gastroenterol Nutr 2008 46 615 618 10.1097/MPG.0b013e31816e213a 18493224

[B5] Vesikari T Van Damme P Giaquinto C Gray J Mrukowicz J Dagan R Guarino A Szajewska H Usonis V European Society for Paediatric Infectious Diseases/European Society for Paediatric Gastroenterology, Hepatology, and Nutrition evidence-based recommendations for rotavirus vaccination in Europe J Pediatr Gastroenterol Nutr 2008 46 Suppl 2 S38 S48 10.1097/MPG.0b013e31816f7a10 18460971

[B6] World Health Organization Meeting of the immunization Strategic Advisory Group of Experts, April 2009–conclusions and recommendations Wkly Epidemiol Rec 2009 84 220 236 19499606

[B7] Vesikari T Karvonen A Puustinen L Zeng SQ Szakal ED Delem A De VB Efficacy of RIX4414 live attenuated human rotavirus vaccine in Finnish infants Pediatr Infect Dis J 2004 23 937 943 10.1097/01.inf.0000141722.10130.50 15602194

[B8] Vesikari T Karvonen A Prymula R Schuster V Tejedor JC Cohen R Meurice F Han HH Damaso S Bouckenooghe A Efficacy of human rotavirus vaccine against rotavirus gastroenteritis during the first 2 years of life in European infants: randomised, double-blind controlled study Lancet 2007 370 1757 1763 10.1016/S0140-6736(07)61744-9 18037080

[B9] Vesikari T Matson DO Dennehy P Van Damme P Santosham M Rodriguez Z Dallas MJ Heyse JF Goveia MG Black SB Safety and efficacy of a pentavalent human-bovine (WC3) reassortant rotavirus vaccine N Engl J Med 2006 354 23 33 10.1056/NEJMoa052664 16394299

[B10] Gimenez-Sanchez F Gado-Rubio A Martinon-Torres F Bernaola-Iturbe E Multicenter prospective study analysing the role of rotavirus on acute gastroenteritis in Spain Acta Paediatr 2010 99 738 742 20096025 10.1111/j.1651-2227.2010.01684.x

[B11] Diez-Domingo J Baldo JM Patrzalek M Pazdiora P Forster J Cantarutti L Pircon JY Soriano-Gabarro M Meyer N Primary care-based surveillance to estimate the burden of rotavirus gastroenteritis among children aged less than 5 years in six European countries Eur J Pediatr 2011 170 213 222 10.1007/s00431-010-1289-1 20842379

[B12] Iturriza-Gomara M Kang G Gray J Rotavirus genotyping: keeping up with an evolving population of human rotaviruses J Clin Virol 2004 31 259 265 10.1016/j.jcv.2004.04.009 15494266

[B13] Kuhne Simmonds M Armah G Asmah R Banerjee I Damanka S Esona M Gentsch JR Gray JJ Kirkwood C Page N New oligonucleotide primers for P-typing of rotavirus strains: strategies for typing previously untypeable strains J Clin Virol 2008 42 368 373 10.1016/j.jcv.2008.02.011 18378188

[B14] Ruuska T Vesikari T Rotavirus disease in Finnish children: use of numerical scores for clinical severity of diarrhoeal episodes Scand J Infect Dis 1990 22 259 267 10.3109/00365549009027046 2371542

[B15] Viala-Danten M Meunier J Arnould B Development and psychometric validation of a new questionnaire measuring the impact of child gastroenteritis on parents. Poster presented at ISPOR, Athens, 8–11 November 2008 Value Health 2008 11 A526 A527

[B16] Forster J Guarino A Parez N Moraga F Roman E Mory O Tozzi AE de Aguileta AL Wahn U Graham C Hospital-based surveillance to estimate the burden of rotavirus gastroenteritis among European children younger than 5 years of age Pediatrics 2009 123 e393 e400 10.1542/peds.2008-2088 19254975

[B17] Senecal M Brisson M Lebel MH Yaremko J Wong R Gallant LA Garfield HA Ableman DJ Ward RL Sampalis JS Measuring the Impact of Rotavirus Acute Gastroenteritis Episodes (MIRAGE): a prospective community-based study Can J Infect Dis Med Microbiol 2008 19 397 404 19436568 10.1155/2008/451540 PMC2663469

[B18] Brisson M Senecal M Drolet M Mansi JA Health-related quality of life lost to rotavirus-associated gastroenteritis in children and their parents: a Canadian prospective study Pediatr Infect Dis J 2010 29 73 75 10.1097/INF.0b013e3181b41506 19907361

[B19] Brooks R EuroQol: the current state of play Health Policy 1996 37 53 72 10.1016/0168-8510(96)00822-6 10158943

[B20] Dolan P Modeling valuations for EuroQol health states Med Care 1997 35 1095 1108 10.1097/00005650-199711000-00002 9366889

[B21] Mast TC Muro-Mercon C Kelly CM Floyd LE Walter EB The impact of rotavirus gastroenteritis on the family BMC Pediatr 2009 9 11 10.1186/1471-2431-9-11 19200366 PMC2649068

[B22] Gimenez-Sanchez F Delgado RA Martinon TF Asensi BF Miranda VM Gomez Llorente JL Alfayate MS Carmona MA Romero GJ Crespo HM Family impact of rotavirus gastroenteritis in children under two years An Pediatr (Barc) 2008 69 515 520 10.1016/S1695-4033(08)75233-0 19128763

[B23] Van der Wielen M Giaquinto C Gothefors L Huelsse C Huet F Littmann M Maxwell M Talayero JM Todd P Vila MT Impact of community-acquired paediatric rotavirus gastroenteritis on family life: data from the REVEAL study BMC Fam Pract 2010 11 22 10.1186/1471-2296-11-22 20230601 PMC2841655

[B24] Huppertz HI Forster J Heininger U Roos R Neumann HU Hammerschmidt T The Parental Appraisal of the Morbidity of Diarrhea in Infants and Toddlers (PAMODI) survey Clin Pediatr (Phila) 2008 47 363 371 10.1177/0009922807310933 18270310

