# Supplementary material for: The impact of childhood acute rotavirus gastroenteritis on the parents’ quality of life: prospective observational study in European primary care medical practices
Source: BMC Pediatr. 2012 May 31;12:58. doi: 10.1186/1471-2431-12-58 (PMC3495402; doi:10.1186/1471-2431-12-58)
Supplement: Additional file 1 — Questionnaire validation. Details of the validation of the questionnaire in DOC format. [file 1471-2431-12-58-S1.doc]

# Additional File 1

**[[To be submitted as a separate file in PDF format]]**

## Questionnaire development

A pilot questionnaire was developed and linguistically validated in Spanish, Polish and Italian. The initial pilot version of the questionnaire consisted of 54 items:

- The child’s symptoms (a total of 24 items):
  - Diarrhoea (items 1 to 3)
  - Vomiting (items 4 to 6)
  - Fever (items 7 to 9)
  - Dehydration (items 10, 10a, 10b)
  - Discomfort (items 11 to 13)
  - Meal (items 14 to 16)
  - Weight loss (items 17 to 19)
  - Medication (item 20)
  - Duration of gastroenteritis (items 21 to 22)
- The child’s behaviour during the gastroenteritis (a total of 8 items – items 23a to 23h)
- Anxiety due to the child’s gastroenteritis (9 items)
- Worries about spread of gastroenteritis (item 24)
- Overall worries (item 25)
- Feelings (items 26a to 26g)
- Daily activities during the child’s gastroenteritis (11 items, items 27 to 37)
- Impact of child’s gastroenteritis on parent’s occupation (2 items, items 38 and 39)

The response choices for each item ranged from 4 to 9 points. Higher scores indicated higher levels of negative feelings.

## Item reduction

The mean number of missing items was 1.43 out of 54 items in the questionnaire, or less than 3% per subject. Items were grouped into three hypothesised scales: signs, emotions, and activities, and multitrait analysis was performed on data from respondents who had at least 50% of items completed in each scale. A total of 10 items from the initial questionnaire were removed because of a high percentage of missing data or low convergent and/or discriminant validity. The hypothesised “signs” scale was split into the final “Symptom Severity” and “Child’s Behaviour” scales, and the hypothesised “emotions” scale was split into the “Parents’ Worries due to Symptoms” and “Parents’ Distress” scales. The hypothesised “activities” scale became the “Impact on Parents’ Daily Activities” scale. The final questionnaire consisted of 44 items in five scales: “Symptom Severity” (13 items); “Child’s Behaviour” (6 items); “Parents’ Worries due to Symptoms” (8 items); “Parents’ Distress” (7 items); and “Impact on Parents’ Daily Activities” (10 items) (Supplementary Table 1).

## Statistical methods

Cronbach’s alpha was used to assess internal consistency, and the Spearman correlation coefficient was used to analyse the relationship between scores and other parameters. Convergent validity was considered satisfactory if the correlation between each item score and its scale was at least 0.40. Discriminant validity was considered satisfactory if each item shared a higher correlation with its own scale than with other scales.

## Psychometric properties

A total of 291 questionnaires provided sufficient data (at least 50% of the items completed) for analysis of psychometric properties. No scale showed evidence of a floor (high percentage of responders at the lowest possible score) or ceiling (high percentage of responders at the highest possible score) effect (Supplementary Table 2). Item convergent and discriminant validity was good for all the scales (Supplementary Table 3), and Cronbach’s alpha ranged from 0.78 to 0.85, indicating high internal consistency reliability (Supplementary Table 3). Scale–scale correlations are presented in Supplementary Table 4. As expected, correlations were high between the “Symptom Severity” and “Parents’ Distress” scores (0.67) and between “Parents’ Distress” and “Impact on Parents’ Daily Activities” (0.63) (Supplementary Table 4). The other correlations ranged from 0.41 to 0.57. These correlations indicated a strong relationship between all the scales, showing consistency but no redundancy.

Supplementary Table 1. Final structure and conceptual contents of the questionnaire

| **Scale** | **Number of items** | **Items** |
| --- | --- | --- |
| Symptom Severity | 13 | Diarrhoea duration  Nappy changes  Vomiting duration  Vomiting frequency  Fever duration  Fever: temperature  Dehydration  Discomfort or pain duration  Discomfort or pain level  Not full meal: duration  Refuses to eat  Weight loss  Weight loss level |
| Parents’ Worries due to Symptoms | 8 | Worry about child’s diarrhoea  Worry about child’s vomiting  Worry about child’s temperature  Worry about child’s dehydration  Worry about child’s discomfort or pain  Worry about child’s lack of appetite  Worry about child’s weight loss  Overall, worry about child’s gastroenteritis |
| Child’s Behaviour | 6 | Cry more than usual  Irritable  Less playful  Exhausted  Less alert  Need extra comforting |
| Impact on Parents’ Daily Activities | 10 | Nights disturbed: frequency  Sleep loss  Leisure activities  Food shopping  Housework  Cares for your child: duration  Help from others: duration  Financial difficulties: loss of income  Financial difficulties: added expenses  Missed paid work duration |
| Parents’ Distress | 7 | Feel upset  Feel helpless  Feel mentally exhausted  Feel physically exhausted  Feel tired  Feel fed up  Break the heart to see the child ill |

Supplementary Table 2. Floor and ceiling effects (n=291)

| **Scale** | **N (%) at floor** | **N (%) at ceiling** |
| --- | --- | --- |
| Symptom Severity | 0 (0.0%) | 0 (0.0%) |
| Parents’ Worries due to Symptoms | 0 (0.0%) | 19 (6.5%) |
| Child’s Behaviour | 0 (0.0%) | 21 (7.2%) |
| Impact on Parents’ Daily Activities | 0 (0.0%) | 2 (0.7%) |
| Parents’ Distress | 1 (0.3%) | 12 (4.1%) |

Supplementary Table 3. Item convergent and discriminant validity and Cronbach’s alpha (n=291)

| **Scale** | **Number of items** | **Range of item-scale correlations** | **Items meeting convergent validity criterion (%)** | **Items meeting discriminant validity criterion (%)** | **Cronbach’s alpha** |
| --- | --- | --- | --- | --- | --- |
| Symptom Severity | 13 | 0.23-0.63 | 62% | 62% | 0.79 |
| Parents’ Worries due to Symptoms | 8 | 0.38-0.61 | 88% | 75% | 0.78 |
| Child’s Behaviour | 6 | 0.58-0.68 | 100% | 100% | 0.85 |
| Impact on Parents’ Daily Activities | 10 | 0.33-0.64 | 90% | 90% | 0.83 |
| Parents’ Distress | 7 | 0.50-0.69 | 100% | 100% | 0.84 |

Supplementary Table 4. Correlation between the scales of the questionnaire (n=291)

|  | **Symptom severity** | **Parents’ worries due to symptoms** | **Child’s behaviour** | **Impact on parents’ daily activities** |
| --- | --- | --- | --- | --- |
| Parents’ worries due to symptoms | 0.67 |  |  |  |
| Child’s behaviour | 0.53 | 0.41 |  |  |
| Impact on parents’ daily activities | 0.57 | 0.53 | 0.50 |  |
| Parents’ distress | 0.44 | 0.48 | 0.50 | 0.63 |
